# Evaluation of Asymmetric Dimethylarginine (ADMA) Levels in Children with Growth Hormone Deficiency

**DOI:** 10.4274/Jcrpe.1182

**Published:** 2014-03-05

**Authors:** Aşan Önder, Zehra Aycan, Cemile Koca, Merve Ergin, Semra Çetinkaya, Sebahat Yılmaz Ağladıoğlu, Havva Nur Peltek Kendirci, Veysel Nijat Baş

**Affiliations:** 1 Dr. Sami Ulus Gynecology and Obstetrics, Children Health and Diseases Training and Research Hospital, Pediatric Endocrinology Clinic, Ankara, Turkey; 2 Atatürk Education and Research Hospital, Department of Biochemistry, Ankara, Turkey

**Keywords:** growth hormone, asymmetric dimethylarginine, child

## Abstract

**Ob­jec­ti­ve**: To investigate serum asymmetric dimethylarginine (ADMA) levels in children with isolated growth hormone deficiency (GHD) and to determine the effect of GH replacement therapy on these levels.

**Methods**: 31 patients diagnosed with isolated GHD and 29 age-and sex-matched healthy children were enrolled in the study. Height, weight and waist circumference were measured in all subjects. Fasting serum insulin-like growth factor-1 (IGF-1), IGF binding protein-3, glucose, insulin and lipid levels were evaluated. Serum ADMA levels were assessed using the enzyme-linked immunosorbent assay technique. The same evaluations were repeated on the 3^rd^ and 6^th^ months of treatment in 28 of the GHD cases.

**Results**: There were no significant differences in ADMA levels between the patient and control groups [0.513±0.130 (0.291-0.820) µmol/L vs. 0.573±0.199 (0.241-1.049) µmol/L]. There was a positive correlation between serum ADMA and HbA1c levels in the control group. In the GHD cases, ADMA levels negatively correlated with high-density lipoprotein levels and positively correlated with low-density lipoprotein levels. There was also a significant increase in ADMA levels in patients receiving GH therapy compared to pre-treatment levels [serum ADMA level, 1.075±0.133 (0.796-1.303) µmol/L at the 3rd month and 0.923±0.121 (0.695-1.159) µmol/L at the 6th month of treatment]. There was a negative correlation between ADMA levels and homeostasis model assessment of insulin resistance values at the 6th month evaluation. There were no relationships between ADMA levels and age, sex, or pubertal state either before or during the treatment.

**Conclusion**: Serum ADMA levels were found to be similar in patients with GHD and in healthy children. However, serum ADMA levels showed a significant increase in GHD patients following GH replacement therapy.

## INTRODUCTION

Asymmetric dimethylarginine (ADMA) is an endogenous inhibitor of nitric oxide synthase (NOS). It is formed by the methylation of arginine, followed by its hydrolysis. 90% of ADMA is metabolized by dimethylaminohydrolase (DDAH) enzymes to citrulline and dimethylamine, while 10% is excreted in the urine without being metabolized. The reduction in NO synthesis following an increase in ADMA levels causes endothelial dysfunction. This means that there is an important relationship between ADMA and atherogenesis. ADMA levels rise in patients with cardiovascular system disorders who manifest clinical symptoms, as well as in patients with diabetes mellitus, multiple organ failure, hyperthyroidism, chronic kidney disease, insulin resistance (IR) or metabolic syndrome and preeclampsia (1,2,3). The importance of monitoring ADMA levels during the pediatric period can better be appreciated in view of the fact that the symptoms of most cardiovascular and renal system disorders that emerge in adulthood begin during childhood.

Although it is known that growth hormone deficiency (GHD) can lead to cardiovascular system dysfunction and dyslipidemia, there are no studies in the literature on ADMA levels in children with GHD. In a study with adults diagnosed with hypopituitarism, Krzyzanowska et al (4) showed that ADMA levels increased independent of GHD and well-recognized risk factors. In another study by Setola et al (5), ADMA levels were observed to decrease following a 6-month GH therapy in adults with GHD and in addition, there was a negative correlation between insulin-like growth factor-1 (IGF-1) and ADMA levels. It is expected that treatment approaches in pediatric applications for ADMA control will be implemented in the future (6).

In the present study, we aimed to determine whether ADMA levels in children with GHD differed from those in healthy children and also to evaluate the effects of GH therapy on serum ADMA levels.

## METHODS

The study was conducted in the Pediatric Endocrinology Clinic at Dr. Sami Ulus Gynecology and Obstetrics, Children’s Health and Disease Training and Research Hospital, between February and August 2012. Approval from the ethics committee and informed consent forms from the parents of the participants were obtained prior to the study. 31 patients who were diagnosed with isolated GHD and scheduled to be started on GH treatment and 29 age-and sex-matched healthy children were enrolled in the study. The patient group consisted of cases with a height standard deviation score (SDS) of ≤-2.5 SDS, a bone age level of 2 years/or more below the chronological age, a growth rate of ≤-1 SDS and who showed insufficient GH responses to two stimulation tests (peak GH <10 ng/mL). Patients with chronic systemic diseases were not included in the study group. Those with a history of any other chronic diseases, those receiving routine medication, as well as those with insufficiency in other anterior pituitary hormones, with syndromic short stature and those who were overweight or obese (relative weight (RW) >110%] were also excluded. Detailed medical histories of all cases were recorded and anthropometric evaluations were carried out. Weight was measured using a SECA brand scale with 0.1 kg sensitivity and a Harpenden stadiometer (Holtain Instruments Ltd., UK) was used for height measurement. All measurements were performed by the same nurse. Height and weight SDS were calculated in accordance with the accepted standards in Turkey (7). Waist circumferences of the participants were also recorded. Waist circumferences were measured, by the same person using a non-stretch measuring tape, as the circumference at a level midway between the iliac crest and the lowest palpable rib. Pubertal staging was determined according to the Tanner and Marshall criteria (8). Morning fasting blood samples were collected from all subjects. The samples were centrifuged at 4000 rpm for 10 minutes. HbA1c, glucose, high-density lipoprotein (HDL), low-density lipoprotein (LDL), triglyceride and total cholesterol measurements were performed using the spectrophotometric technique with a Beckman Coulter LX20 (Synchron Systems) autoanalyzer. Insulin values were determined with the chemiluminescence immunoassay technique using an Immulite 2000 immunoassay system (Siemens). Homeostasis model assessment of IR (HOMA-IR) was calculated using the following formula: fasting insulin (µU/mL) x fasting glucose (mmol/L) / 22.5 (9). Percentile values for lipid concentrations were calculated by age and sex in accordance with reported reference values (10). IGF-1 and IGF binding protein-3 (IGFBP-3) levels were measured with the chemiluminescence technique using the Immulite 2000 immunoassay system (Siemens) and SDS values were calculated by age and sex in accordance with reported reference values (11). Collected blood samples were stored in -40˚C prior to measurement of ADMA levels, which was performed using the enzyme-linked immunosorbent assay technique and ImmunDiagnostik (Bensheim, Germany) kits.

Patients with GHD were started on 0.2 mg/kg/week of GH following pre-treatment assessment and serum ADMA levels and other parameters (e. g., serum lipid, glucose and insulin levels) were evaluated on the 3^rd^ and 6^th^ months of treatment in 28 cases that were followed up regularly. Three cases were removed from the study due to failure to observe the treatment schedule. 

**Statistical Analyses**

Statistical analyses were performed using the Statistical Package for Social Sciences (SPSS) version 15 for Windows. Descriptive statistics were expressed as mean±SD for variables with a normal distribution and as median (minimum-maximum) for variables without a normal distribution. Normal distribution was assessed by the Shapiro-Wilk test. The significance of the differences between the means was evaluated using student’s t-test and that between median values was evaluated using Mann-Whitney U-test. The significance of the difference between two pairs for variables (prior to GH therapy and in the 3rd and 6th months of treatment) was assessed using the paired-samples t-test. Pearson’s correlation test was used to evaluate the relationship between two variables when a normal distribution was present and Spearman’s correlation test was used in the absence of a normal distribution. Statistical significance was set at p<0.05.

## RESULTS

Thirty-one patients with isolated GHD and 29 healthy children who served as controls were included in the study. The mean age of the patient group was 12.5±1.8 (8.5-16.5) years. There were 19 males and 12 females in the patient group; 11 cases were at prepubertal stage and 20 were pubertal. The mean age of the control group was 11.5±2.1 (7.3-16.6) years. The control group consisted of 18 males and 11 females; 8 cases were at prepubertal stage, the remaining 21 were pubertal. There were no differences between the study group with isolated GHD and the control group in terms of age, sex and pubertal state. Height SDS and weight SDS were significantly lower in GHD patients as compared to the control group. However, there were no differences between the two groups in terms of RW and waist circumference.

Mean serum ADMA level was 0.513±0.130 (0.291-0.820) µmol/L in the patient group and 0.573±0.199 (0.241-1.049) µmol/L in the control group (p=0.165). There were no significant differences in terms of serum ADMA levels between the patient and control groups, either in the prepubertal or in the pubertal stages (Table 1). Moreover, there was no significant difference in terms of serum ADMA levels between the sexes among both groups. There was no relationship of ADMA with height, weight, height SDS, weight SDS, RW, IGF-1/IGFBP-3 and their SDS values in the two groups. 

**Figure 1:**
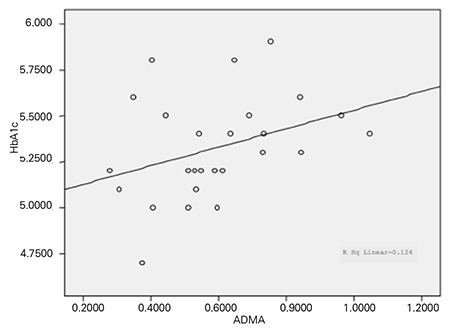
The correlation between asymmetric dimethylarginine (ADMA) and HbA1c in the control group

Evaluation of the relationship between serum ADMA levels and carbohydrate metabolism revealed no correlation between ADMA and insulin, HbA1c, glucose levels, or HOMA-IR values in the patient group. There was a positive correlation (p=0.033; correlation coefficient=0.420) between ADMA and HbA1c in the control group (Figure 1). However, there was no correlation between ADMA and insulin, glucose, or HOMA-IR in the control group.

**Figure 2:**
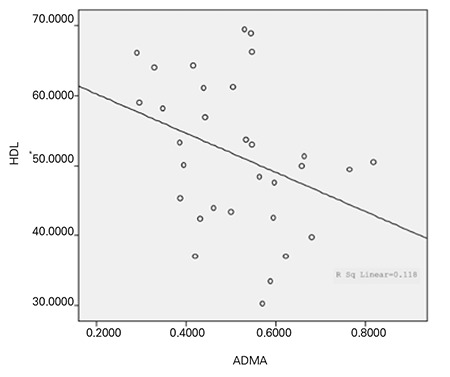
The correlation between asymmetric dimethylarginine (ADMA) and high density lipoprotein (HDL) in the patient group before treatment

**Figure 3:**
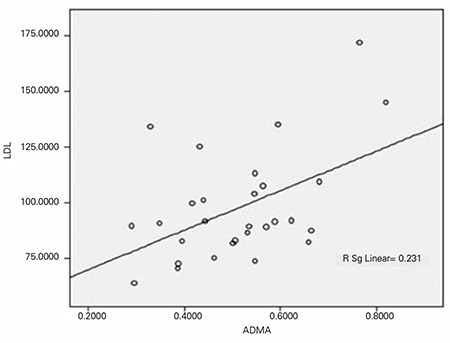
The correlation between asymmetric dimethylarginine (ADMA) and low density lipoprotein (LDL) in the patient group before treatment

Evaluation of the relationship between ADMA and lipid levels in the patients revealed a negative correlation between ADMA and HDL (p=0.028; correlation coefficient=-0.394) and a positive correlation between ADMA and LDL (p=0.040; correlation coefficient=0.383) (Figures 2 and 3). There was no relationship between ADMA and HDL or LDL values in the control group. There also was no statistically significant relationship between ADMA and HDL/LDL percentiles, total cholesterol and triglyceride vvalues or their percentiles amongst either of the groups.

**Figure 4:**
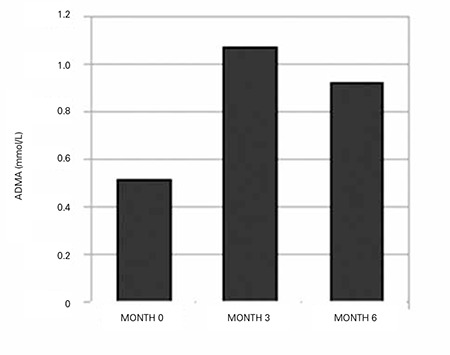
Serum asymmetric dimethylarginine (ADMA) levels before and during treatment in the patient group

**Figure 5:**
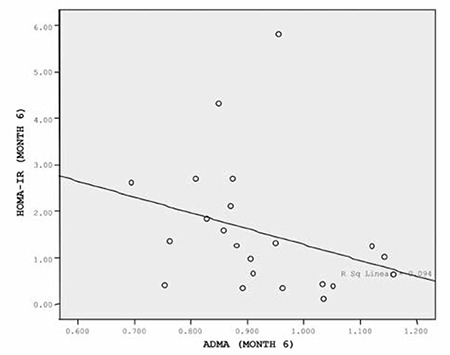
The correlation between asymmetric dimethylarginine (ADMA) and homeostasis model assessment of insulin resistance (HOMA-IR) at the end of the 6-month treatment

Statistically significant increases in ADMA levels were found in the evaluation of 3^rd^ and 6^th^ month data from 28 cases who were on treatment and were being followed up regularly (p=0.000). Mean serum ADMA level was 1.075±0.133 (0.796-1.303) µmol/L at the end of the 3^rd^ month and 0.923±0.121 (0.695-1.159) µmol/L at the end of the 6^th^ month of treatment (Figure 4). There was a negative correlation between ADMA levels and HOMA-IR values at the 6^th^ month evaluation (p=0.016; vcorrelation coefficient=-0.506; Figure 5). On the other hand, no significant relationships were found between ADMA and fasting glucose, insulin, lipids, IGF-1, or IGFBP-3 during the treatment period. There also were no changes in ADMA levels as related to age, sex and pubertal stage.

## DISCUSSION

The increase in ADMA levels causes a decrease in NO levels, leading to endothelial dysfunction and an increased risk of cardiovascular disease and mortality (12). In adult GHD patients, dyslipidemia, elevated blood pressure, abnormal body composition, increased weight, increase in inflammation indicators and increased coagulability predispose individuals to cardiovascular diseases and early atheromatosis (13). However, factors that lead to these negative outcomes in patients with GHD remain unclear. To date, there are only a few studies, all conducted on adult patients, which investigate the relationship between GHD and ADMA. In a study on adult patients with hypopituitarism, ADMA levels were reported to be higher in the patients than in healthy controls. The elevated ADMA levels in these patients were found to be similar in the GHD and non-GHD groups (4). In another study conducted on 31 GHD patients, a significant increase in cyclic guanosine 3’,5’-monophosphate (cGMP) levels and a significant decrease in ADMA levels following a 6-month GH replacement therapy were found. These same authors also reported that post-treatment IGF-1 levels positively correlated with cGMP levels and arginine/ADMA ratios and negatively correlated with ADMA levels (5). In a different study, Thum et al (14) administered GH therapy to 16 healthy adults for a duration of 10 days and evaluated their NO indicators following this treatment. Similar to previous studies, this study showed that there was a positive correlation between IGF-1 and cGMP levels. Moreover, it was also reported that there was a significant decrease in ADMA levels following treatment. An increase in cGMP levels has also been reported in mice that were given IGF-1 and lastly, when the IGF-1 receptors were blocked, it was reported that the cGMP and ADMA values were unaffected by GH therapy. Thus, GH therapy increases systemic NO bioavailability through IGF-1 (14). All of the above studies have been conducted on adults.

In the present study, the ADMA levels were similar in the GHD and control groups (p=0.165). As expected, the IGF-1 and IGFBP-3 levels were lower in the GHD cases, but this difference was not reflected in the ADMA values. Also, in the current study, a relationship could not be established between the ADMA levels and age, sex and pubertal stage neither in the GHD nor in the control groups. Similarly, in a study by Ayer et al (15) conducted on 405 healthy children, no gender differences in ADMA levels were reported. In another study investigating 68 obese and 68 healthy children, ADMA levels were also found to be independent of the participants’ sex. In a study with adolescents, ADMA levels were found to be negatively correlated with age both in obese and healthy participants (16). To our knowledge, there are no previous studies examining the relationship between ADMA levels and puberty in children. In the present study, we found no difference between prepubertal and pubertal serum ADMA levels neither in healthy children nor in children with GHD.

Assessment of anthropometric data obtained in the present study revealed that there was no correlation between ADMA levels and weight, height, RW, or waist circumference. Similarly, in the study by Ayer et al (15) on healthy children, the authors reported that ADMA levels did not correlated with waist circumference and body mass index. In a different study, Gruber et al (16) reported that ADMA levels were significantly increased in obese adolescents than in normal weight participants. In the same study, in the obese group and in the whole study group, a negative correlation was reported between height and ADMA. In many studies on adults, obesity has been shown to trigger an increase in ADMA levels secondary to dyslipidemia and hyperinsulinism (17,18). In the present study, overweight and obese cases were therefore excluded.

Evaluation of the cases in terms of carbohydrate metabolism revealed that there was no difference between fasting blood sugar, HbA1c and HOMA-IR levels in either group. ADMA levels were not correlated with fasting glucose, insulin, HbA1c and HOMA-IR values in the patient group. There was also no relationship between ADMA levels and fasting glucose, insulin, or HOMA-IR values in the healthy control group. However, the positive correlation determined between the HbA1c and ADMA levels in healthy children was remarkable. All studies show that IR and diabetes mellitus cause impairment in NO production or in its bioavailability in the endothelium. All manifestations of hyperinsulinism, hyperglycemia, hyperlipidemia and oxidative stress that may be observed in diabetes are responsible for endothelium dysfunction (3). However, the relationship between insulin-glucose metabolism and ADMA is quite complex. Plasma ADMA levels may show a decrease with increased cationic amino acid transporter (CAT) activity or an increase with decreased DDAH activity (19). In a study conducted on 174 children and adolescents, which included 85 type 1 diabetes mellitus patients and 89 healthy controls, it was found that ADMA levels were decreased in type 1 diabetes patients and that there was an inverse relationship between ADMA and HbA1c levels. ADMA levels were lower in diabetes patients without vasculopathy than in diabetes patients with vasculopathy. In the control group, glucose levels were correlated with ADMA levels and it was reported that the glucose levels had significant predictive values for ADMA levels. The authors commented that the higher levels of ADMA in healthy children and adolescents did not reflect a damaging mechanism, but one that protects against the oxygen radicals that may be released as a result of excessive NO production followed by oxidative stress (20). In the present study, even though there was no statistically significant difference, ADMA levels were higher in the healthy control group. Interestingly, despite the correlation between ADMA and HbA1c in the control group, there was no relationship between glucose and ADMA levels.

In the current study, it was quite important and significant to find that ADMA levels were negatively correlated with serum HDL levels and positively correlated with LDL levels, even in such a young age group with GHD. There was no relationship between ADMA and lipid values in the healthy control group. In a study on 58 hypercholesterolemic and 37 healthy children, Hasanoglu et al (20) reported that ADMA levels were significantly higher in the group with elevated cholesterol compared to the healthy children. A correlation between total cholesterol/triglyceride and ADMA could not be established. In the same study, similar to the present one, ADMA levels were positively correlated with LDL levels. Jehlicka et al (21) reported that ADMA levels were higher in children with familial hypercholesterolemia than in children with type 1 diabetes or in healthy controls. In a study conducted with 405 healthy children, the increase in ADMA levels was found to be positively correlated with the thickness of carotid intima-media. In the same study, no relationship was found between ADMA and lipid levels (15).

Contrary to our expectations, the current study revealed that ADMA levels were significantly increased in GHD children receiving GH treatment. This increase in serum ADMA levels, which got close to the levels observed in healthy children, can be interpreted as an attempt to protect from oxygen radicals, just as Huemer et al (22) indicated. The present study involved a period of 6 months of GH therapy. In this regard, in order to better evaluate treatment responses, there may be a need for studies with longer follow-up durations. In our subjects, we found no relationship between the ADMA levels and fasting glucose, insulin, IGF, or lipid profiles during the treatment. Also, the ADMA levels did not correlate with age, sex, weight, height, RW and pubertal state in the assessments performed on the 3^rd^ and 6^th^ months. Therefore, increased ADMA levels could not be associated with increasing age and presence of dyslipidemia during the follow-up period. On the 3^rd^ month, a significant increase was noted in HOMA-IR levels and even though there was no correlation between the two, ADMA levels were also increased in the same period. This might indicate a relationship between ADMA and IR in cases who received GH therapy. Intracellular ADMA levels were not evaluated in the current study. It is possible that GH therapy might decrease intracellular ADMA levels while increasing serum ADMA levels.

In conclusion, the present study, which we believe is the first study investigating the serum ADMA levels in pediatric patients with GHD and in healthy children of similar ages, showed that the GH therapy led to an increase in ADMA levels. The negative correlation between serum ADMA and HDL levels and the positive correlation between serum ADMA and LDL levels observed in the GHD cases might indicate that these children may have problems with lipid metabolism in their advanced ages. Evaluating ADMA levels in tissues rather than in the serum in children might yield more realistic results. Further studies with greater numbers of subjects and longer follow-up periods are needed.

## Figures and Tables

**Table 1:**
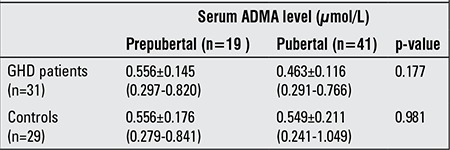
Pubertal and prepubertal serum asymmetric dimethylarginine (ADMA) levels in patients with growth hormone deficiency (GHD) and in the controls
